# Paradoxical effect of rapamycin on inflammatory stress-induced insulin resistance *in vitro* and *in vivo*

**DOI:** 10.1038/srep14959

**Published:** 2015-10-09

**Authors:** Ping Yang, Yunfei Zhao, Lei Zhao, Jun Yuan, Yao Chen, Zac Varghese, John F. Moorhead, Yaxi Chen, Xiong Z. Ruan

**Affiliations:** 1 Centre for Lipid Research, Key Laboratory of Molecular Biology on Infectious Diseases, Ministry of Education, the Second Affiliated Hospital, Chongqing Medical University, Chongqing, 400016, China; 2 School of Biological & Chemical Engineering, Chongqing University of Education, Chongqing, 400067, China; 3 John Moorhead Research Laboratory, Centre for Nephrology, University College London (UCL) Medical School, Royal Free Campus, London, NW3 2PF, UK

## Abstract

Insulin resistance is closely related to inflammatory stress and the mammalian target of rapamycin/S6 kinase (mTOR/S6K) pathway. The present study investigated whether rapamycin, a specific inhibitor of mTOR, ameliorates inflammatory stress-induced insulin resistance *in vitro* and *in vivo*. We used tumor necrosis factor-alpha (TNF-α) and interleukin-6 (IL-6) stimulation in HepG2 hepatocytes, C2C12 myoblasts and 3T3-L1 adipocytes and casein injection in C57BL/6J mice to induce inflammatory stress. Our results showed that inflammatory stress impairs insulin signaling by reducing the expression of total IRS-1, p-IRS-1 (tyr632), and p-AKT (ser473); it also activates the mTOR/S6K signaling pathway both *in vitro* and *in vivo*. *In vitro*, rapamycin treatment reversed inflammatory cytokine-stimulated IRS-1 serine phosphorylation, increased insulin signaling to AKT and enhanced glucose utilization. *In vivo*, rapamycin treatment also ameliorated the impaired insulin signaling induced by inflammatory stress, but it induced pancreatic β-cell apoptosis, reduced pancreatic β-cell function and enhanced hepatic gluconeogenesis, thereby resulting in hyperglycemia and glucose intolerance in casein-injected mice. Our results indicate a paradoxical effect of rapamycin on insulin resistance between the *in vitro* and *in vivo* environments under inflammatory stress and provide additional insight into the clinical application of rapamycin.

Insulin resistance is the central component of metabolic syndrome and an important pathophysiological factor in the initiation and development of type 2 diabetes (T2DM), atherosclerosis (AS) and non-alcoholic fatty liver disease (NAFLD)^1^,^2^. Insulin resistance is defined as a clinical state in which insulin-responsive tissues (including liver, muscle and adipose tissue) are insensitive to normal or elevated insulin levels and subsequently trigger a series of pathophysiological events. Classic insulin signaling that involves insulin receptor, insulin receptor substrates (IRS), phosphatidylinositol-3-kinase (PI3K) and the protein kinase AKT plays a central role in the metabolic actions of insulin in many types of cells and tissues^3^,^4^. In pathophysiological states, such as inflammatory stress, free fatty acid loading and oxidative stress, uncontrolled inhibitory serine phosphorylation of IRS-restrained tyrosine phosphorylation and hijacked insulin signaling^5^,^6^. It is now well-accepted that the dysregulation of IRS phosphorylation is a major parameter resulting in insulin resistance; however, the precise cellular mechanism is largely unknown.

Low-grade systemic inflammation is a feature of obesity and insulin resistance^7^. Since Hotamisligil and colleagues first showed that pro-inflammatory cytokine tumor necrosis factor-alpha (TNF-α) was able to induce insulin resistance in four different rodent models of obesity and diabetes^8^, further inflammatory markers and mediators including C-reactive protein (CRP) and interleukin-6 (IL-6) were reported to be closely associated with T2DM^9^,^10^. The molecular mechanisms by which inflammation causes insulin resistance have been intensively investigated in the last decade. It has been demonstrated that adipose-derived pro-inflammatory cytokines (adipokines), such as TNF-α, IL-6, monocyte chemoattractant protein-1 (MCP-1) and serum amyloid A (SAA), may act locally in autocrine and paracrine manners or play a systemic role in modulating insulin actions^11^,^12^,^13^,^14^. Numerous investigations indicate that inflammation or individual cytokines inhibit insulin signaling by several mechanisms, such as serine-phosphorylation of IRS-1, induction of suppressor of cytokine signaling 3 (SOCS-3), and the activation of c-Jun NH2-terminal kinase (JNK) or inhibitor kappa B kinase β/nuclear transcription factor kappa B (IKKβ/NF-κB) pathway in insulin-target tissues^12^,^15^,^16^,^17^,^18^. However, much is still unknown regarding the molecular mechanism of inflammation leading to serine-phosphorylation of IRS-1.

Recently, our previous study and studies by others demonstrated that inflammation can activate the mammalian target of the rapamycin (mTOR) pathway^19^,^20^,^21^. The mTOR/S6 kinase (S6K) pathway is a critical signaling component in the development of obesity-linked insulin resistance^22^,^23^. Sustained activation of the mTOR/S6K pathway by nutrients or prolonged insulin treatment promotes insulin resistance through increased IRS-1 serine phosphorylation, leading to a reduction in IRS-1 function and impaired activation of the PI3K/AKT pathway, thereby creating a negative feedback loop of insulin action. Whether inflammation leads to phosphorylation of IRS-1 at the serine site through the mTOR pathway remains to be discussed.

Rapamycin is a very selective inhibitor of the mTOR/S6K pathway, with no inhibitory activity toward other known kinases, even at concentrations in the high nanomolar range^24^. Accordingly, the inhibition of mTOR by rapamycin was found to attenuate serine phosphorylation of IRS-1, restore insulin action on the PI3K/AKT pathway and prevent the insulin-resistant effects of excess nutrients on insulin-mediated glucose transport in muscle and adipose cells^25^,^26^,^27^,^28^. Paradoxically, it appears that a significant side effect of chronic rapamycin treatment is deranged glucose metabolism^29^. Chronic administration of rapamycin substantially impairs glucose tolerance and insulin action under conditions of excess nutrients^30^. However, the effects of rapamycin on insulin action and glucose metabolism under the condition of inflammatory stress remain totally unknown *in vivo*.

The current study was undertaken to investigate whether inflammatory stress impairs insulin signaling by activating the mTOR/S6K pathway in cultured cells (HepG2 cells, C2C12 myoblasts and 3T3-L1 adipocytes) and C57BL/6J mice. Moreover, we aimed to investigate the effects of rapamycin on inflammatory stress-induced insulin resistance and glucose metabolism disorder both *in vitro* and *in vivo*. The different effects of rapamycin on insulin resistance between *in vivo* and *in vitro* environments under inflammatory stress are further discussed in this study.

## Results

### Rapamycin recovered inflammatory cytokine-induced impairment of glucose uptake and consumption *in vitro*

**Figure 1:**
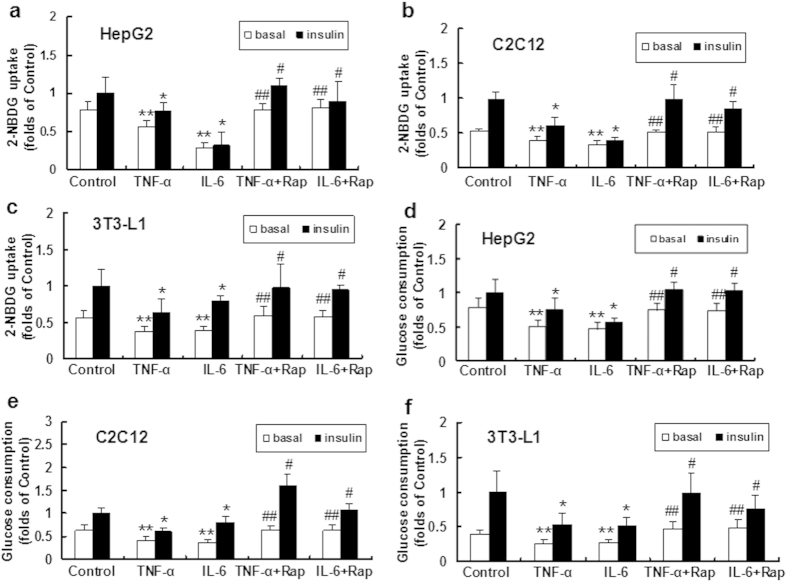
Effects of inflammatory stress and rapamycin on glucose utilization *in vitro*. HepG2 cells, differentiated C2C12 myoblasts and 3T3-L1 adipocytes were incubated in serum-free medium for 24 h. The medium was then replaced by fresh serum-free medium containing 0.04 mmol/L palmitic acid alone or with TNF-α (25 ng/ml) or IL-6 (20 ng/ml) in the absence or presence of rapamycin (10 ng/ml) for 24 h. Glucose uptake (**a**–**c**) was determined with a fluorescent D-glucose analog, 2NBDG, after treatment with or without insulin. Glucose consumption (**d**–**f**) was measured as described in the Materials and Methods section and normalized by cell protein. The results are shown as the mean ± SD (n = 6). **P < 0.05 versus control (basal), *P < 0.05 versus control (insulin), ^##^P < 0.05 versus inflammatory cytokine treated group (basal), ^#^P < 0.05 versus inflammatory cytokine treated group (insulin).

HepG2 cells, C2C12 myoblasts and 3T3-L1 adipocytes were treated with TNF-α or IL-6 to induce inflammatory stress. Our data showed that glucose (2-NBDG) uptake and glucose consumption were decreased in the TNF-α and IL-6-treated groups compared with controls in the three types of cell lines in the absence and presence of insulin (Fig. 1a–f), suggesting that inflammatory stress impaired glucose utilization *in vitro*.

We further analyzed the effect of rapamycin on glucose metabolism in the three types of cell lines. Glucose uptake and consumption were improved in the rapamycin plus TNF-α or IL-6 groups compared with cells treated with TNF-α or IL-6 alone in the absence and presence of insulin (Fig. 1a–f), suggesting that rapamycin ameliorates inflammatory stress-induced insulin resistance *in vitro*.

### Rapamycin improved inflammatory cytokine-induced defects in insulin signaling *in vitro*

**Figure 2:**
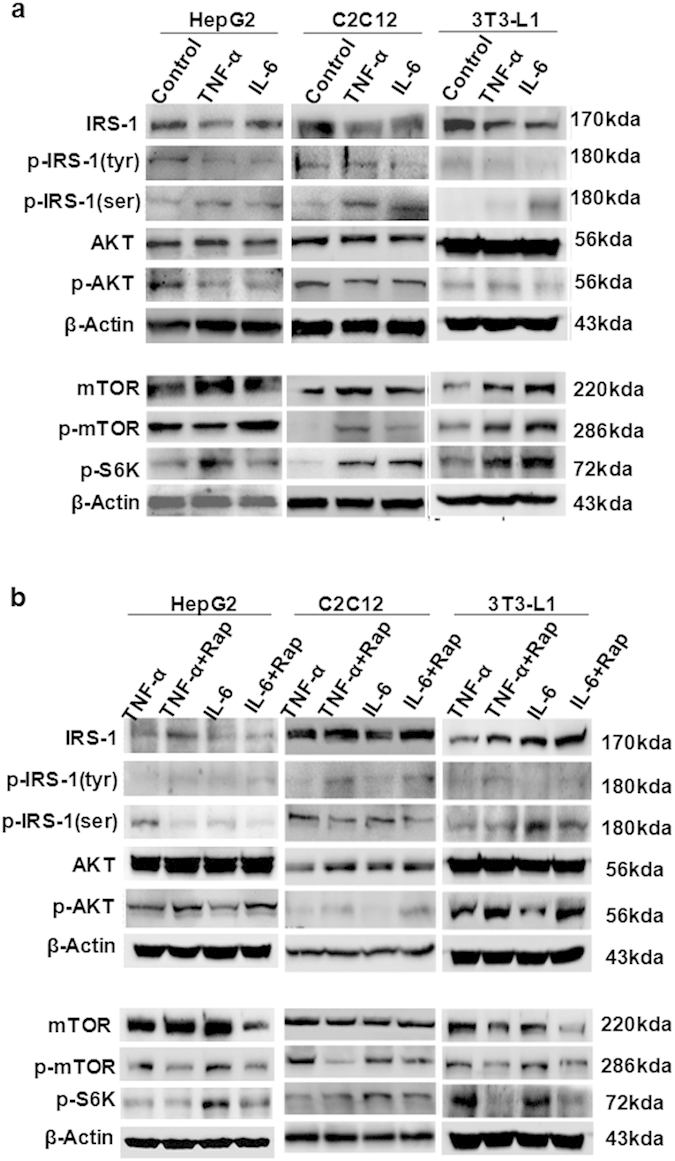
Effects of inflammatory stress and rapamycin on protein expression of insulin signaling pathway *in vitro*. HepG2 cells, C2C12 myoblasts and 3T3-L1 adipocytes were treated with insulin prior to harvest. (**a**) IRS1, p-IRS1 (tyr), and p-AKT, proteins of insulin signaling, were downregulated in the TNF-α- or IL-6-treated group. The proteins involved in the mTOR/S6K pathway were upregulated in the presence of inflammatory cytokines. (**b**) Under inflammatory stress, rapamycin inhibited the protein expression of mTOR, p-mTOR and p-S6K, while increasing the proteins involved in insulin signaling.

The total protein levels of IRS-1 and tyrosine phosphorylated IRS-1 were reduced in the TNF-α and IL-6-treated groups compared to controls. In addition, serine AKT phosphorylation was downregulated in the inflammatory cytokine loading group compared with controls; however, no significant change was observed in total AKT expression (Fig. 2a).

We also observed that serine phosphorylated IRS-1 was increased after TNF-α or IL-6 loading (Fig. 2a), implying that inflammatory stress impaired insulin signaling. As the potential effector molecules involved in insulin resistance, the levels of mTOR, phosphorylated mTOR and downstream phosphorylated S6K were increased in the TNF-α and IL-6-treated groups (Fig. 2a), suggesting that the mTOR/S6K pathway may be involved in insulin resistance under inflammatory stress.

Next, we evaluated the effects of rapamycin on impaired insulin signaling induced by inflammatory stress in three types of cell lines. Rapamycin inhibited the inflammatory stress induced p-mTOR and p-S6K expression, reduced the levels of serine phosphorylated IRS-1, and increased total IRS-1 and tyrosine phosphorylated IRS-1 expression (Fig. 2b). Quantitative densitometry analysis of western blot was also performed (see Supplementary Fig. S1 online). These results suggested that rapamycin improves the insulin signaling pathway under inflammatory stress.

### Casein injection induced chronic systemic and local inflammation in C57BL/6J mice

**Figure 3:**
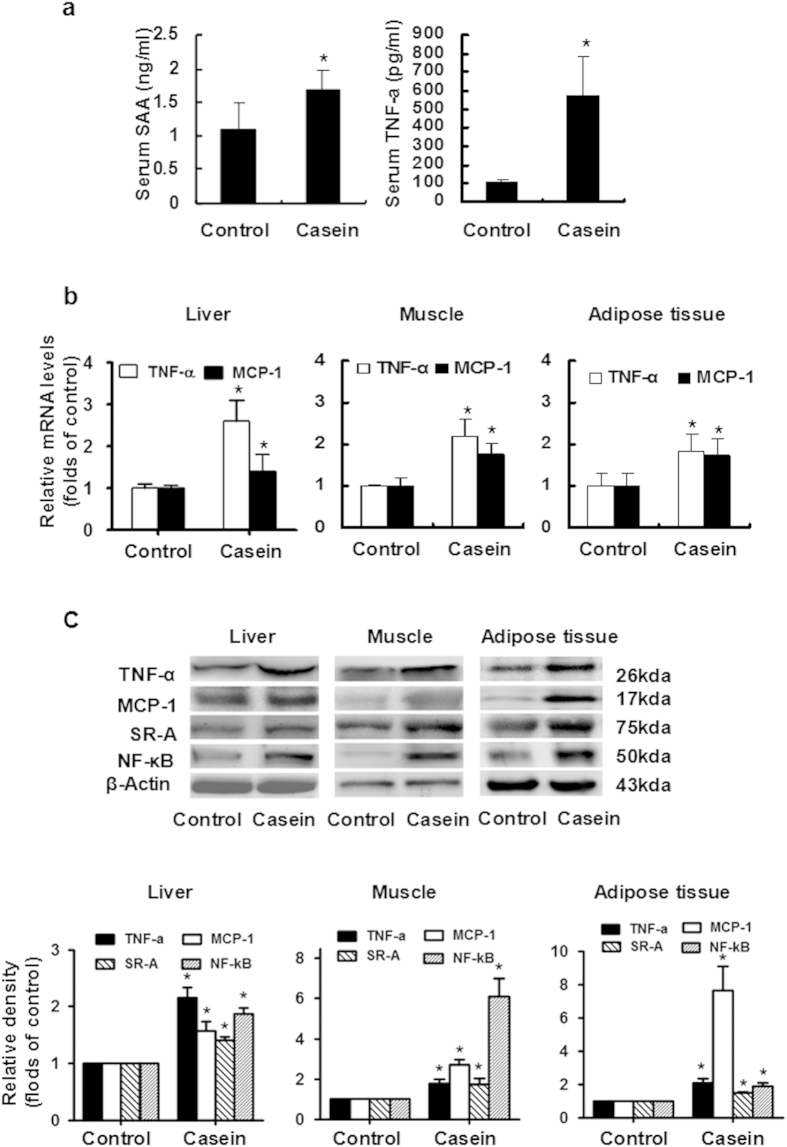
Casein injection induced chronic systemic inflammation *in vivo*. The C57BL/6J mice fed with high-fat diet (HFD) were randomly assigned to receive a subcutaneous injection of normal saline (control group) or casein (casein group). (**a**) The levels of SAA and TNF-α in the serum of casein-injected mice were significant higher. The mRNA (**b**) and protein (**c**) levels of TNF-α and MCP-1 in the liver, muscle and adipose tissues increased in the casein group, which was parallel to the upregulation of SR-A and NF-kB protein in the liver, muscle and adipose tissue. The histogram represents the mean ± SD (n = 3) of the densitometric scans for protein bands, normalized by comparison with β-actin, and expressed as folds of control. *P < 0.05 versus control.

Chronic low-grade systemic inflammation was induced in C57BL/6J mice using a combination of a high-fat diet and a subcutaneous injection of 10% casein on alternate days for 14 weeks. There were significant increases in the SAA and TNF-α concentration in the serum of casein-injected mice compared with controls (Fig. 3a). We also measured local inflammatory cytokine production in the liver, skeletal muscle and adipose tissue of C57BL/6J mice. The mRNA expression of TNF-α and MCP-1, and the protein expression of TNF-α, MCP-1, scavenger receptor type A (SR-A) and NF-κB in the liver, muscle and adipose of casein-injected mice were significantly increased compared with those of control mice (Fig. 3b,c). These results suggested that inflammatory stress was successfully induced in C57BL/6J mice.

### Chronic inflammation induced insulin resistance and lipid disorder *in vivo*

**Figure 4:**
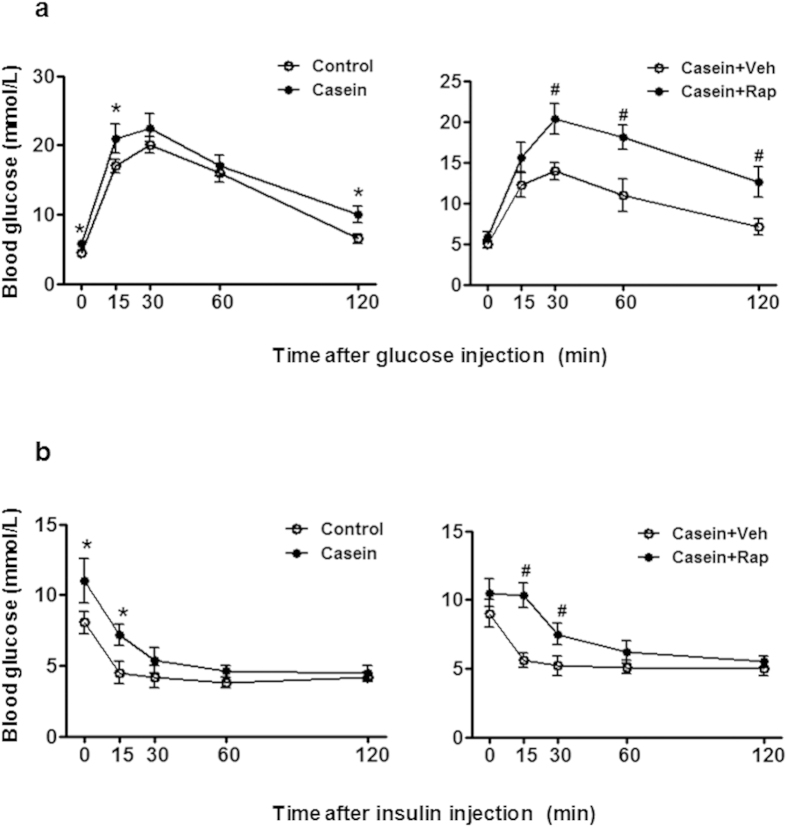
Effects of chronic inflammation and rapamycin on glucose and insulin tolerance *in vivo*. The C57BL/6J mice fed with a high-fat diet (HFD) were randomly assigned to receive a subcutaneous injection of normal saline, casein, casein plus vehicle and casein plus rapamycin. (**a**) Glucose tolerance tests (glucose-stimulated blood glucose concentrations, IGTT) performed after 14 weeks of treatment. (**b**) Insulin tolerance tests (insulin-stimulated blood glucose concentrations, IITT) performed at the end of the experiments. The results represent the mean ± SD (n = 6). * P < 0.05 versus control. ^#^P < 0.05 versus casein plus vehicle group.

The fasting glucose levels were significantly increased in casein-injected mice compared to controls. After a glucose challenge, the blood glucose concentration in the inflamed group was persistently higher than the control group, especially at the 120-minute time point of the glucose challenge (Fig. 4a left), suggesting that chronic inflammation induced glucose intolerance in mice. After insulin loading, blood glucose levels decreased less in the casein-treated group (Fig. 4b left), suggesting that chronic inflammation reduced insulin sensitivity *in vivo*. We also analyzed the lipid levels in C57BL/6J mice. As shown in Table 1, triglyceride (TG) and free fatty acid (FFA) levels in the liver and muscle were significantly increased in casein-treated mice, while serum and adipose tissue lipid content was unchanged/unaffected, suggesting that chronic systemic inflammation led to ectopic lipid deposition in non-adipose tissue.

### Rapamycin improved IRS-1/AKT signaling pathway but aggravated insulin resistance *in vivo*

**Figure 5:**
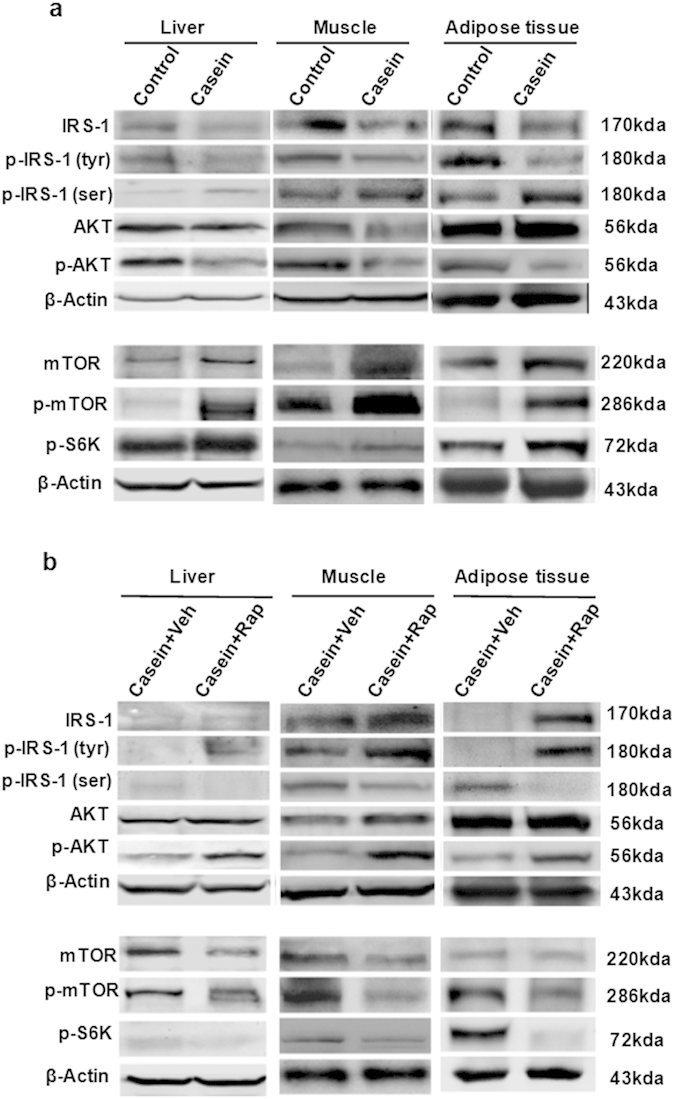
Effects of chronic inflammatory and rapamycin on protein expression of insulin signaling *in vivo*. (**a**) IRS1, p-IRS1 (tyr), and p-AKT, proteins of insulin signaling, were downregulated in casein group. The proteins involved in the mTOR/S6K pathway were upregulated in the presence of casein. (**b**) Under inflammatory stress, rapamycin inhibited the protein expression of mTOR, p-mTOR and p-S6K, while increasing the proteins involved in insulin signaling.

Inflammatory stress inhibited the levels of total IRS-1, tyrosine phosphorylated IRS-1, p-AKT and increased the levels of serine phosphorylated IRS-1, which is parallel to the overactivation of the mTOR/S6K pathway in the liver, muscle and adipose tissue of C57BL/6J mice (Fig. 5a). Rapamycin inhibited the inflammation-induced upregulation of p-mTOR, p-S6K and serine p-IRS1, and increased total IRS-1, tyrosine p-IRS1 and p-AKT levels in liver, muscle and adipose of C57BL/6J mice (Fig. 5b). Quantitative densitometry analysis of western blot was also performed (see Supplementary Fig. S2 online). The results suggested that inflammatory stress impaired insulin signaling probably via the activation of the mTOR/S6K pathway *in vivo*, which can be blocked by rapamycin.

Unexpectedly, glucose (Fig. 4a right) and insulin (Fig. 4b right) tolerance tests from C57BL/6J mice showed that rapamycin aggravated glucose intolerance under inflammatory stress. However, TG and FFA levels in the liver and muscle of C57BL/6J mice were markedly decreased in the presence of rapamycin (Table 1), suggesting that rapamycin could ameliorate lipid accumulation induced by inflammation in non-adipose tissue.

### Rapamycin treatment enhanced hepatic gluconeogenesis in inflamed mice

**Figure 6:**
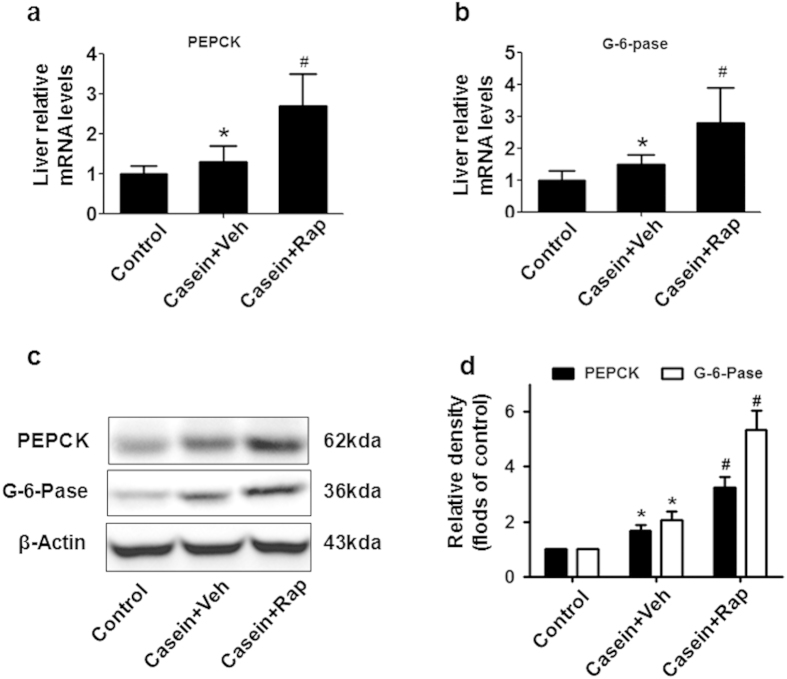
Rapamycin treatment enhanced hepatic gluconeogenesis. (**a**) PEPCK and (**b**) G-6-Pase mRNA expression in the liver of C57BL/6J mice. The graphs depict mRNA expression in the liver of target genes corrected for the expression of β-actin as the housekeeping gene. (**c**) Representative western blotting of PEPCK and G-6-pase proteins in the liver. (**d**)The histogram represents the mean ± SD (n = 3) of the densitometric scans for protein bands from three experiments, normalized by comparison with β-actin, and expressed as folds of control. *P < 0.05 versus control, ^#^P < 0.05 versus casein plus vehicle.

Because rapamycin had an opposite effect on glucose metabolism under inflammatory stress *in vitro* and *in vivo*, we focused on the contribution of the liver to the glucose intolerance of rapamycin-treated mice. Chronic inflammation upregulated the mRNA (Fig. 6a,b) and protein (Fig. 6c,d) expression of two key gluconeogenic enzymes, phosphoenolpyruvate carboxykinase (PEPCK) and glucose-6-phosphatase (G-6-Pase), in the liver. Notably, rapamycin treatment resulted in even higher expression of PEPCK and G-6-Pase than the casein-treated group (Fig. 6a–d).

### Rapamycin induced pancreatic β-cell dysfunction and apoptosis in inflamed mice

**Figure 7:**
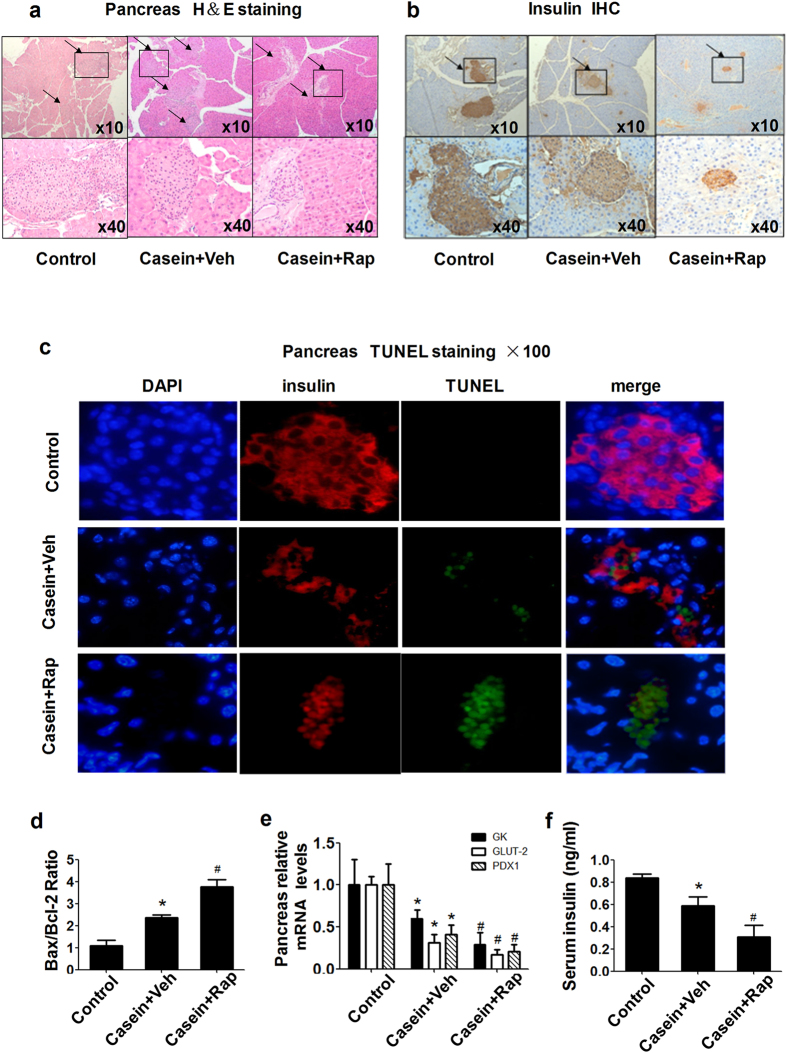
Rapamycin induced pancreatic β-cell dysfunction and apoptosis *in vivo*. (**a**) Hematoxylin–eosin staining in the pancreas. (**b**) Insulin immunohistochemistry. Magnification, 10× (top panels); 40× (bottom panels). (**c**) Apoptosis *in situ*. Apoptosis of insulin-expressing cells on islet sections was determined by the TUNEL assay. Representative examples of pancreatic islets stained by immunofluorescence for insulin (red), marker of cell apoptosis TUNEL (green), and nuclear stain DAPI (blue) imaged at 100× in casein plus vehicle (upper) and casein plus rapamycin (lower). (**d**) Effects of rapamycin on the mRNA expression of Bax/Bcl2 in the pancreas of C57BL/6J mice. (**e**) Effects of rapamycin on β-cell gene expression. The expression of GK, GLUT2 and PDX-1 in islets was determined by real-time quantitative RT-PCR using β-actin as an internal standard. (**f**) Serum insulin concentrations in C57BL/6J mice were analyzed using a mouse-specific insulin ELISA kit. The results represent the mean ± SD (n = 6). *P < 0.05 versus control, ^#^P < 0.05 versus casein plus vehicle.

In accordance with our previous study, chronic inflammation exacerbated pancreatic β-cell dysfunction and apoptosis. In the rapamycin-treated group, the islet size and insulin content were further decreased (Fig. 7a,b), suggesting that rapamycin impairs β-cell function in casein-injected mice. In addition, we found that rapamycin increased β-cell apoptosis (Fig. 7c) and upregulated pancreatic Bax/Bcl2 mRNA expression (Fig. 7d). To gain further insight into the mechanism underlying rapamycin-mediated impairment in pancreatic islets, we determined the expression of key participants in β-cell functional integrity, namely pancreatic duodenal homeobox-1 (PDX-1), glucokinase (GK), and glucose transporter 2 (GLUT2). Rapamycin significantly reduced pancreas PDX-1, GLUT2 and GK mRNA levels (Fig. 7e), and the serum insulin concentration in the rapamycin treatment group was lower (Fig. 7f). Together these results clarify why rapamycin ameliorates impaired insulin signaling induced by inflammation, but does not improve the overall glucose tolerance *in vivo*.

## Discussion

Chronic systemic inflammation plays an important role in the pathogenesis of multiple metabolic disorders, including insulin resistance, T2DM, NAFLD, AS, obesity and dyslipidemia. In this study, we used two important inflammatory cytokine, TNF-α and IL-6, to induce inflammatory stress in HepG2 cells, C2C12 myoblasts and 3T3-L1 adipocytes. We verified the results from *in vitro* study using an *in vivo* model of chronic inflammation by subcutaneous casein injection in C57BL/6J mice fed a HFD. Casein injection is an established method for the induction of chronic systemic inflammation in mouse models^31^,^32^. Compared to other sole cytokine-treated models or local inflammation models, casein injection increased multiple cytokine levels in the serum, which is more likely to mimic the chronic systemic inflammatory state observed in patients with inflammatory stress^33^. In this model, serum SAA and TNF-α levels were significantly increased, and TNF-α, MCP-1, SR-A and NF-kB in the liver, muscle and adipose tissue were upregulated, confirming that we established a successful model of chronic inflammation (Fig. 3). We further evaluated the effects of inflammation on glucose metabolism *in vitro* and *in vivo*. TNF-α or IL-6 downregulated insulin-stimulated glucose uptake and consumption in three types of cell lines, and casein injection decreased glucose and insulin tolerance in C57BL/6J mice, indicating that the inflammatory stress promoted insulin resistance (Figs 1 and 4).

mTOR/S6K has received much attention recently because of its central role in modulating cell proliferation and affecting tumorigenesis as well as its involvement in insulin resistance^34^,^35^. From a collective body of work, mTOR/S6K signaling has emerged as a key player in insulin resistance resulting from a variety of conditions, including excessive nutrients and hyperinsulinemia. To investigate the molecular link between inflammatory stress and insulin resistance, we investigated the role of the mTOR/S6K pathway on insulin signaling. Rapamycin (also known as Rapamune® (sirolimus)) is a specific inhibitor of the mTOR pathway. Here, we investigated the contribution of rapamycin to insulin resistance and glucose metabolism under inflammatory stress both *in vitro* and *in vivo*. In this study, we showed that rapamycin improved the attenuated IRS1/PI3K/AKT insulin signaling pathway induced by inflammatory stress, increased the levels of total IRS-1 and tyrosine phosphorylated IRS-1 and decreased serine phosphorylation of IRS-1 both *in vitro* and *in vivo* (Figs 2b and 5b). Our results suggested that inflammatory stress induced serine phosphorylation of IRS-1, impairing insulin signaling probably via activation of the mTOR/S6K pathway *in vitro* and *in vivo*. There may be some limitations to the pharmacological approach of using rapamycin to block mTOR signaling. Rapamycin inhibits the activity of the two mTOR complexes. Inhibition of mTORC1 occurs immediately following binding of rapamycin to FK506-binding protein-12 (FKBP12), whereas prolonged exposure to rapamycin is required to block formation of nascent mTORC2. Therefore, genetic attenuation of mTORC1 signaling or deletion of the mTORC1 substrate S6K may help to strengthen our conclusion. Further, assessing the dose-dependent effect of inflammatory factors on mTOR signaling activities as well as conducting a correlation analysis may provide more evidence to support this conclusion.

In recent years, rapamycin has been used as a key component of the immunosuppressive regimen in clinical organ transplantation. It was suggested that rapamycin may hold promise as a drug for diabetes treatment. However, it was found that organ transplant patients treated with rapamycin have a higher prevalence of glucose intolerance than others. This may suggest that rapamycin disrupts glucose metabolism under certain conditions, such as abnormal immune response or inflammation induced by organ transplantation. Here, the beneficial effect of rapamycin on glucose uptake and consumption *in vitro* was contradictory to the aggravated glucose intolerance and reduced insulin sensitivity observed *in vivo* under inflammatory stress (Fig. 4).

Given the severe glucose intolerance and hyperglycemia in rapamycin-treated mice under inflammation, we first evaluated the contribution of the liver to this phenomenon. Liver gluconeogenesis is driven by the availability of gluconeogenic substrates and the activity of two key gluconeogenic enzymes, PEPCK and G-6-Pase. In the re-fed state, insulin suppresses gluconeogenesis by transcriptional downregulation of PEPCK and G-6-Pase^36^. Our data revealed that casein treatment results in higher levels of PEPCK and G-6-pase in the mouse liver. Rapamycin further enhanced hepatic gluconeogenesis by upregulating PEPCK and G-6-pase expression in casein-injected mice (Fig. 6), which is consistent with previous reports that chronic rapamycin treatment increased hepatic gluconeogenesis in rats^29^.

We subsequently investigated the effects of rapamycin on pancreatic insulin secretion. PDX-1 acts in β-cells as a house-keeping transcription factor for insulin gene expression^37^,^38^. GK and GLUT2 are two key components of the glucose-sensing machinery responsible for glucose-inducible insulin release^38^,^39^,^40^. We showed that rapamycin significantly reduced pancreas PDX-1, GLUT2 and GK mRNA levels accompanied by β-cell apoptosis (Fig. 7). Serum insulin concentrations in the rapamycin treatment group were lower, suggesting that rapamycin impairs pancreatic β-cell function and reduces insulin synthesis and secretion. It has been shown that mTOR plays a critical role in the regulation of β-cell mass. Conversely, the inhibition of mTOR by rapamycin causes loss of β-cell function and viability^38^,^41^. This increases our understanding of why rapamycin was beneficial to impaired insulin signaling *in vitro* but further exacerbated glucose intolerance under conditions of inflammatory stress *in vivo*.

Abnormal lipid metabolism is one of the features of metabolic syndrome. Elevated FFA has been shown to impair insulin action and to be a risk factor for the development of T2DM. We have previously demonstrated that rapamycin plays important roles in controlling cholesterol homeostasis^19^,^20^. In this study, we showed that rapamycin treatment ameliorates chronic inflammation-induced disordered lipid metabolism in non-adipose tissue. Rapamycin treatment lowered TG and FFA deposition, especially in the liver and muscle of casein-injected mice (Table 1).

Taken together, this study suggests that inflammatory stress induces the dysregulated phosphorylation of IRS-1 and impairs insulin signaling probably by activating the mTOR/S6K pathway *in vitro* and *in vivo*. Rapamycin has the potential to improve insulin signaling and reduce lipid accumulation in the liver induced by chronic low-grade systemic inflammation, but it also has a negative effect on β-cell function and hepatic gluconeogenesis, which may cause hyperglycemia. Therefore, the overall negative effect of rapamycin on glucose homeostasis is due to a combination of these factors. A method to harness the beneficial effects of rapamycin while maintaining β-cell function and inhibiting hepatic glucose output could potentially lead to a stream of novel medications for the treatment of diabetes.

## Methods

### Cell culture and induction of differentiation

HepG2 cells were cultured in growth medium containing Dulbecco’s modified Eagle’s medium (DMEM)/high-glucose medium, 10% fetal calf serum, 2 mmol/l glutamine, 100 U/ml penicillin, and 100 μg/ml streptomycin. Mouse C2C12 myoblasts were maintained in DMEM/ high-glucose medium with 10% fetal calf serum, 2 mmol/l glutamine, 100 U/ml penicillin, and 100 μg/ml streptomycin at a 70–80% confluency. To initiate differentiation, cells were allowed to reach 100% confluency, and the medium was changed to DMEM containing 2% horse serum and changed every 2 days. Full differentiation, with myotube fusion and spontaneous twitching was observed at 5 days. 3T3-L1 preadipocytes were cultured in growth medium containing DMEM/ high-glucose medium with 10% calf serum, 2 mmol/l glutamine, 100 U/ml penicillin, and 100 μg/ml streptomycin. Two-day post-confluent (day 0) cells were introduced to differentiation medium with DMEM/ high-glucose containing 10% fetal bovine serum (FBS), 10 μg/ml insulin (Sigma-Aldrich, St. Louis, MO, USA), 1 μM dexamethasone (Sigma) and 0.5 mM 3-isobutyl-1-methylxanthine (Sigma) until day 2. Cells were fed with DMEM/ high-glucose supplemented with 10% FBS and 10 μg/ml insulin for 2 days followed every other day with DMEM/ high-glucose containing 10% FBS. All experiments were carried out in serum-free DMEM medium containing 0.2% fatty acid-free bovine serum albumin (Sigma), and the cells were subjected to 0.04 mmol/l palmitic acid (PA, Sigma) or inflammatory stress by loading 25 ng/mL TNF-α or 20 ng/ml IL-6 (Peprotech Asia, Rehovot, Israel) in the absence or presence of 10 ng/ml rapamycin (914.19 mol wt, code AY-22989-39, Wyeth Pharmaceutics) for 24 h at 37 °C with 5% CO_2_.

### Animal model

Animal care and experimental procedures were performed with approval from the Animal Care Committee of Chongqing Medical University in accordance with the National Institutes of Health Guide for the Care and Use of Laboratory Animals (NIH publication number 8023, revised 1978). Eight-week-old male C57BL/6J mice were fed a Western diet (Harlan, TD88137) containing 21% fat and 0.15% cholesterol for 14 weeks. The mice were randomly assigned to receive subcutaneous injections of 0.5 milliliter (ml) normal saline or 0.5 ml 10% casein every other day. To investigate the effect of rapamycin, C57BL/6J mice with a combination of a high-fat diet and subcutaneous injection of 10% casein were randomly assigned to receive either a subcutaneous injection of 0.2 ml vehicle or 0.2 ml rapamycin (2 mg/kg body weight). The injections were performed every other day, and the mice were culled 14 weeks after the first injection. At termination, blood samples were taken for lipid profile and cytokine assay and the tissues were collected for further detection.

### Measurement of insulin signaling

For *in vitro* experiments examining insulin signaling, HepG2 cells were harvested after 100 nM insulin stimulation for 15 min, and differentiated C2C12 and 3T3-L1 cells were harvested after 100 nM insulin stimulated for 30 min. For *in vivo* biochemical analysis of insulin signaling, another set of mice in each group was fasted overnight and injected intraperitoneally with insulin at a dose of 5 mU/g body weight (Sigma, St. Louis, MO, US) for 10 min before adipose tissues, liver and skeletal muscle were taken and snap-frozen in liquid nitrogen immediately after resection and stored at −80 °C.

### Serum analysis

Serum tumor necrosis factor-alpha (TNF-α), serum amyloid A protein (SAA) and free fatty acid (FFA) concentrations were measured by enzyme-linked immunosorbent assay (ELISA) kits (HuaMei Biotech, Wu Han, China). Glucose and insulin levels in the serum were detected using the enzyme coupling colorimetric method and ELISA kit, respectively.

### Glucose uptake

Glucose uptake was performed as described previously^42^, with some modification using 2-NBDG (2-[N-(7-nitrobenz-2-oxa-1,3-diazol-4-yl)amino]-2-deoxy- d-glucose; Invitrogen, USA). In brief, after serum starvation for 24 h, the cells were treated with an inflammatory mediator with or without rapamycin at 37 °C for 24 h. Cells were washed three times with wash buffer (20 mM HEPES, pH 7.4, 140 mM NaCl, 5 mM KCl, 2.5 mM MgSO4, and 1 mM CaCl2) then incubated in buffer transport solution (wash buffer containing 10 mM 2-NBDG with or without 100 nmol/L insulin). The 2-NBDG uptake kinetics of cells were measured prior to the irradiation experiment by incubating with buffer transport solution at 37 °C for 20 min. Cell protein content was determined following cell lysis in 1 M NaOH. 2-NBDG uptake was expressed as picomoles per minute per milligram of protein.

### Glucose consumption

After serum starvation for 24 h, cells were treated with inflammatory mediator or rapamycin (containing 100 nmol/L insulin or not) at 37 °C for 24 h. For analysis of glucose concentrations before and after the 24-hour treatment, the medium was spun down in a centrifuge column and subjected to glucose analysis in a GOPOD kit (Rongsheng Biotech, Shanghai, China) according to the manufacturer’s instructions.

### Western blotting

Cytoplasmic and nuclear proteins were extracted from liver, skeletal muscle and white adipose tissue using a commercial kit. Sample proteins were separated by sodium dodecyl sulfate polyacrylamide gel electrophoresis (SDS-PAGE) in a Bio-Rad Mini Protean apparatus and were then transferred to a PVDF membrane. The membranes were blocked with 5% (w/v) non-fat dried milk and incubated with primary antibodies [anti-SR-A, anti-TNF-α, anti-MCP-1, anti-NF-kB, anti-IRS1, anti-p-IRS1 (tyr632), anti-p-IRS1 (ser270), anti-AKT, anti-p-AKT (ser473), anti-mTOR, anti-p-mTOR (S2448), anti-p-S6K(thr421/ser424), anti-β-actin, Santa Cruz Biotechnology, Inc., Santa Cruz, CA, USA)], followed by incubation with a secondary horseradish peroxidase conjugated antibody. Finally, detection procedures were performed using ECL Advance Western Blotting Detection kit (Amersham Bioscience, Piscataway, NJ, US). Band intensity volumes were measured with ImageJ software.

### Quantitative measurement of intracellular TG and FFA

Quantitative measurements of TG and FFA were performed using commercial kits (Jiancheng, Nanjing, China and HuaMei Biotech, Wu Han, China). Briefly, samples were collected and lipids were extracted by addition of 1 ml solvents (TG: heptane/isopropanol = 2/3.5, FFA: chloroform/heptane/methanol = 5/5/1). The lipid phase was collected and vacuum dried. The concentrations of TG and FFA were analyzed using standards and normalized using total protein from tissues.

### Glucose tolerance tests

Before the glucose tolerance tests, mice were starved overnight but allowed free access to water. Glucose tolerance was tested by the intraperitoneal injection of 2 mg D-glucose/g body weight (Sigma). Blood glucose concentrations were determined in blood taken from the cut tail tip before glucose administration and 15, 30, 60, and 120 minutes after the administration. The glucose concentrations were determined using an ACCU-CHEK Advantage blood glucose meter (Roche, Mannheim, Germany).

### Insulin Tolerance Tests

Prior to the insulin tolerance tests, mice were starved for 4 hours but allowed free access to water. Insulin tolerance was measured by intraperitoneal injection of 1 mU insulin/g body weight (Sigma, St. Louis, US). The glucose concentrations in blood were determined using an ACCU-CHEK Advantage blood glucose meter (Roche, Mannheim, Germany), which were taken from the cut tail tip, before and 15, 30, 60, and 120 minutes after the administration of insulin.

### Histology

Pancreas from C57BL/6J mice were sequentially fixed, dehydrated, infiltrated and cut into 5-μm paraffin-embedded tissue sections. Sections were stained with hematoxylin-eosin (HE).

### Immunohistochemistry

Sections were cut from embedded pancreatic slices and deparaffinized in dimethyl benzene. The immunohistochemistry procedures were set up according to the manufacturer’s instructions (Zsbio, Beijing, China). Sections were blocked using 3% hydrogen peroxide and 10% serum and then incubated with primary antibody (anti-insulin, Bioss, Beijing, China). Avidin anti-rabbit antibody served as the secondary antibody followed by horseradish peroxidase anti-avidin antibody. Horseradish peroxidase activity was detected using a DAB solution (Zsbio, Beijing, China). Finally, the reaction was stopped, and sections were counterstained with hematoxylin.

### β-cell apoptosis

Free 3-hydroxy strand breaks resulting from DNA degradation were detected using the terminal deoxynucleotidyl transferase-mediated dUTP nick-end labeling (TUNEL) technique according to the manufacturer’s instructions (DeadEnd™ Fluorometric TUNEL System, Promega, USA). Apoptosis in pancreatic islet was assessed by scoring the number of cells with pyknotic nuclei after DAPI staining. To detect the β-cells, 5-μm slices of paraffin-embedded tissue were incubated with rabbit anti-insulin antibody, followed by detection using the fluorescently labeled secondary antibody. The samples were immediately evaluated by fluorescence microscopy (ZEISS, Germany) for positively stained apoptotic nuclei.

### Real-time Reverse Transcription Polymerase Chain Reaction

Total RNA were isolated from liver, skeletal muscle, white adipose tissue and pancreas homogenates from C57BL/6J mice using Trizol reagent (Takara Life Technologies, Carlsbad, Japan). Real-time reverse transcription polymerase chain reaction (PCR) was performed in a Bio-Rad Sequence Detection System (Hercules, US) using SYBR Green dye (Applied Biosystems Inc, Foster City, US) according to the manufacturer’s protocol.

All the primers were designed by Primer Express Software V2.0 (Applied Biosystems). The following oligonucleotide primers were used for TNF-α (F:5′-CAGCCGATGGGTTGTACCTT-3′, R:5′-GGCAGCCTTGTCCCTTGA-3′). MCP-1 (F:5′-GTCTGTGCTGACCCCAAGAAG-3′, R:5′-TGGTTCCGATCCAGGTTTTTA-3′). Bax (F:5′-GGCCTTTTTGCTACAGGGTTT-3′, R:5′-GTGTCTCCCCAGCCATCCT-3′). Bcl2 (F:5′-CCTGTGGATGACTGAGTACCTGAA-3′, R:5′- CTACCCAGCCTCCGTTATCCT-3′). PDX-1 (F:5′-CGCGTCCAGCTCCCTTT-3′, R:5′-CCACGCGTGAGCTTTGGT-3′). GLUT2 (F:5′-TGGAAGGATCAAAGCAATGTTG-3′, R:5′-CATCAAGAGGGCTCCAGTCAA-3′). GK (F:5′-GCTTTTGAGACCCGTTTTGTG-3′, R:5′-GCCTTCGGTCCCCAGAGT-3′). PEPCK (F:5′-ATCTCCTTTGGAAGCGGATATG-3′, R:5′-CGCAACGCAAAGCATTTCTT-3′). G6pase (F:5′-TCGGCCTATCTCGGGTCTT-3′, R:5′-GCCGCCCAACACTTGGT-3′). β-actin (F:5′-CGATGCCCTGAGGCTCTTT-3′, R:5′-TGGATGCCACAGGATTCCAT-3′). To normalize expression data, β-actin was used as an internal control gene.

### Statistical analysis

Results are presented as means ± SD (n = 6). In all experiments, data were evaluated for statistical significance using one-way ANOVA analysis of variance followed by Q-test. A difference was considered significant if the P value was less than 0.05.

## Additional Information

**How to cite this article**: Yang, P. *et al.* Paradoxical effect of rapamycin on inflammatory stress-induced insulin resistance *in vitro* and *in vivo*. *Sci. Rep.* **5**, 14959; doi: 10.1038/srep14959 (2015).

## Supplementary Material

Supplementary Figure

## Figures and Tables

**Table 1:** The lipid level in the serum, liver, muscle and adipose of C57BL/6J mice fed with HFD.

	Serum		Liver		Muscle		Adipose	
	TG (mmol/L)	FFA (μg/ml)	TG (μg/mg protein)	FFA (ng/mg protein)	TG (μg/mg protein	FFA (ng/mg protein)	TG (mg/mg protein)	FFA (ng/mg protein)
Control	4.62 ± 0.58	2.31 ± 0.55	69.55 ± 11.10	0.12 ± 0.04	36.64 ± 3.37	0.49 ± 0.14	2.42 ± 0.22	12.60 ± 4.79
Casein	4.28 ± 0.23	2.59 ± 0.10	112.01 ± 11.89*	0.31 ± 0.05*	50.17 ± 5.70*	0.67 ± 0.12*	2.19 ± 0.24	11.79 ± 0.96
Casein+ Vehicle	4.41 ± 1.02	1.85 ± 0.30	187.89 ± 38.01	0.45 ± 0.17	52.17 ± 6.09	0.51 ± 0.05	2.89 ± 0.81	17.94 ± 4.86
Casein+ Rapa	5.22 ± 1.88	1.58 ± 0.18	91.56 ± 42.25^§^	0.14 ± 0.05^§^	35.23 ± 11.19^§^	0.39 ± 0.01^§^	1.82 ± 0.36^§^	18.45 ± 2.06

TG and FFA levels in the serum, liver, muscle and adipose tissue of C57BL/6J mice fed with HFD were measured as described in Materials and Methods, and results represent the mean  ±  SD (n = 6). *P < 0.05 versus Control. ^§^P < 0.05 versus Casein + Vehicle.
