# Supplementary material for: Paradoxical effect of rapamycin on inflammatory stress-induced insulin resistance in vitro and in vivo
Source: Sci Rep. 2015 Oct 9;5:14959. doi: 10.1038/srep14959 (PMC4598825; doi:10.1038/srep14959)
Supplement: Supplementary Figure [file srep14959-s1.pdf]

Paradoxical effect of rapamycin on inflammatory stress-induced insulin resistance in vitro and in vivo

Ping Yang[1], Yunfei Zhao[2], Lei Zhao[1], Jun Yuan[1], Yao Chen[1], Zac Varghese[3], John F. Moorhead[3], Yaxi Chen[1*], and Xiong Z. Ruan[1,3]

[1] Centre for Lipid Research, Key Laboratory of Molecular Biology on Infectious Diseases, Ministry of Education， the Second Affiliated Hospital, Chongqing Medical University, Chongqing, 400016, China

[2] School of Biological & Chemical Engineering, Chongqing University of Education, Chongqing, 400067, China

[3] John Moorhead Research Laboratory， Centre for Nephrology, University College London (UCL) Medical School, Royal Free Campus, London, NW3 2PF, UK

*Correspondence: Dr Y Chen, Centre for Lipid Research, Key Laboratory of Molecular Biology on Infectious Diseases, Ministry of Education, the Second Affiliated Hospital of Chongqing Medical University, Chongqing, 400016, China (e-mail: chenyaxi@cqmu.edu.cn)

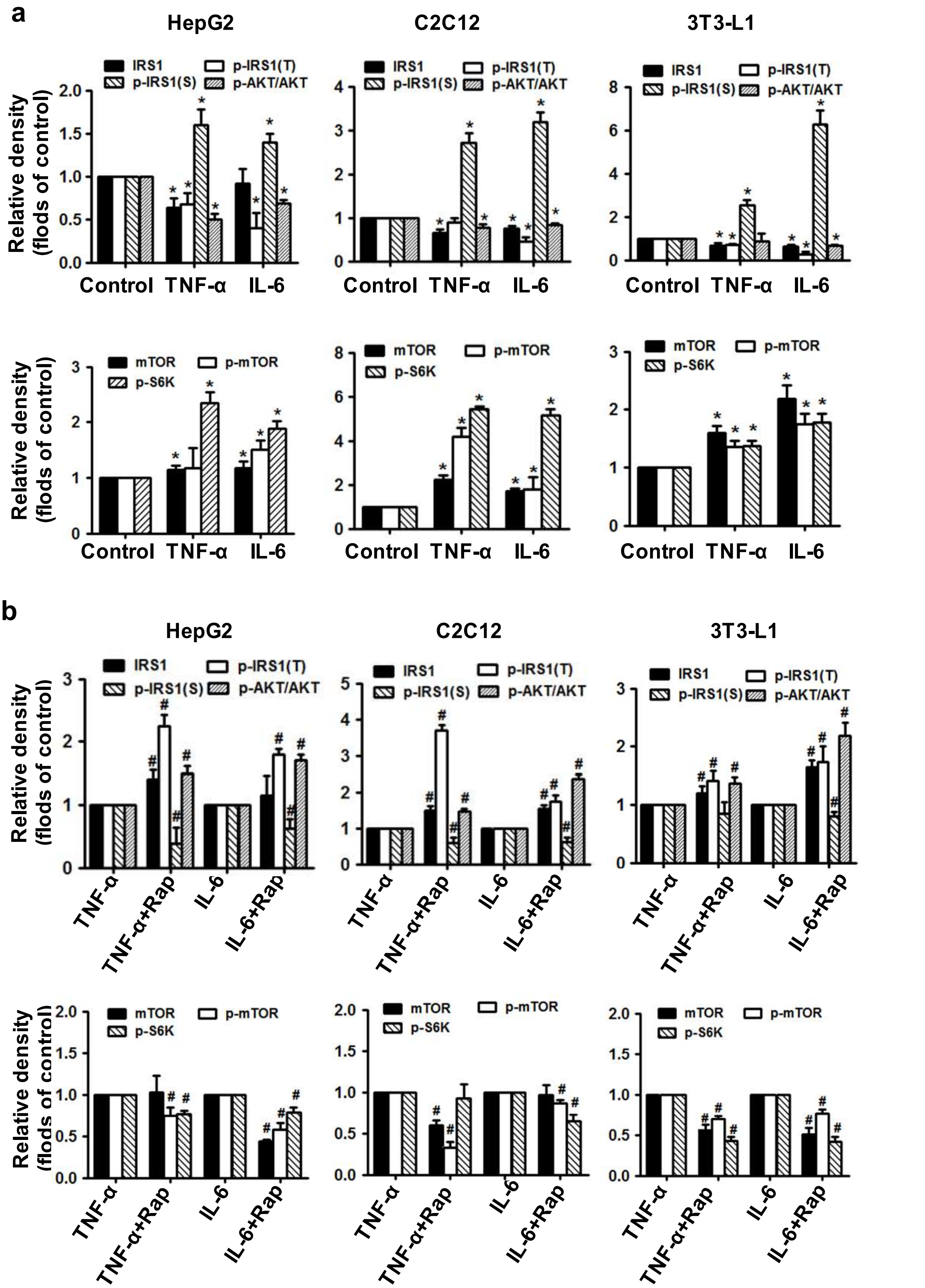

Supplementary Figure S1. Relative protein levels of IRS1, p-IRS1 (tyr), p-IRS1 (ser), p-AKT/AKT, mTOR, p-mTOR, p-S6K in cells treated with TNF-α or IL-6 in the absence (a) or presence of rapamycin (b) were detected by western bolt. The histogram represents the mean ± SD (n=3) of the densitometric scans for protein bands from three experiments, normalized by comparison with β-actin.* $P < 0.05$ versus control, # $P<0.05$ versus the inflammatory cytokine treated group.

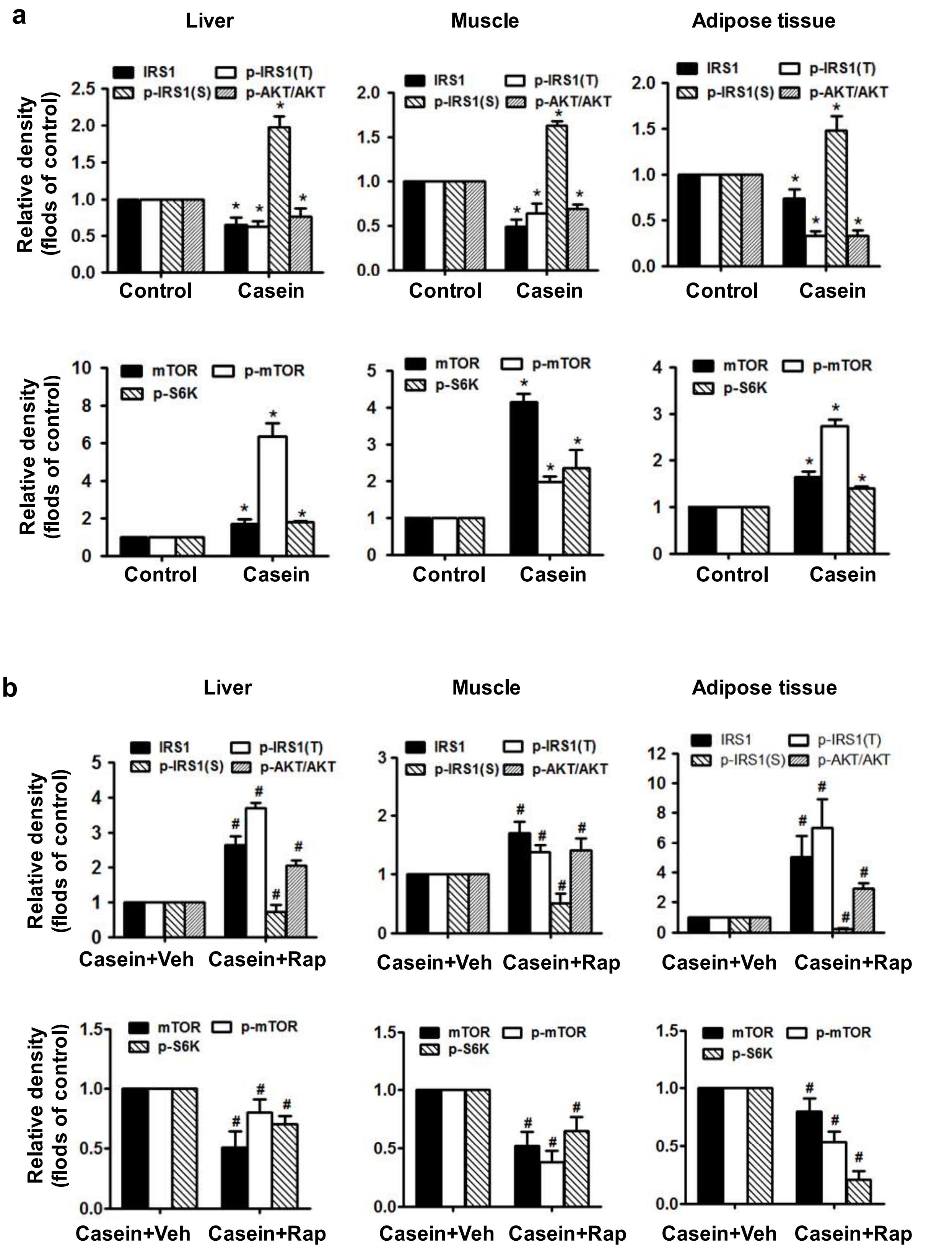

Supplementary Figure S2. Effects of casein (a) and rapamycin (b) on protein levels of IRS1, p-IRS1 (tyr), p-IRS1 (ser), p-AKT/AKT, mTOR, p-mTOR, p-S6K were detected by western bolt. The histogram represents the mean ± SD (n=3) of the densitometric scans for protein bands from three experiments, normalized by comparison with β-actin.* $P < 0.05$ versus control, [#] $P<0.05$ versus casein plus vehicle.
